# Molecular detection and genomic characterization of porcine circovirus 3 in pigs from Northeast China

**DOI:** 10.1186/s12917-018-1634-6

**Published:** 2018-10-26

**Authors:** Zhuo Ha, Chang-zhan Xie, Jin-feng Li, Shu-bo Wen, Ke-long Zhang, Fu-long Nan, He Zhang, Ying-cheng Guo, Wei Wang, Hui-jun Lu, Ning-yi Jin

**Affiliations:** 1 College of Veterinary Medicine, Northeast Agricultural University, Harbin, 150030 China; 2 Institute of Military Veterinary medicine, Academy of Military Medical Sciences, Changchun, 130122 China; 3 College of Animal Science and Technology, Jilin Agricultural University, Changchun, 130118 China; 4 Jilin Fengman area Animal Prevention and Control Center, Jilin, 132013 China; 5 College of Veterinary Medicine, Jilin University, Changchun, 130012 China; 6 College of Animal Science and Technology, Guangxi University, Nanning, 530004 China; 7 Jiangsu Co-Innovation Center for the Prevention and Control of Important Animal Infectious Disease and Zoonoses, Yangzhou University, Yangzhou, 225009 China

**Keywords:** PCV3, Northeast China, Co-infection, Complete genome, Replicase protein

## Abstract

**Background:**

First identified in the United States in 2016, porcine circovirus type 3 (PCV3) is a newly emerging porcine circovirus exhibiting a wide range of clinical syndromes, which may be associated with the pathogenicity observed in pigs.

**Results:**

The aim of this study was to identify and characterize the full genome sequence of PCV3 strains circulating in Northeast China. Herein, 105 lung samples isolated from sick pigs in Northeast China during 2018 were analyzed for PCV3. Using PCR, the total PCV3-positive rate was 33.3% (35/105), with rates of 17.8% (8/45), 66.7% (10/15), and 37.8% (17/45) in Heilongjiang, Jilin, and Liaoning province, respectively. Additionally, our findings showed that PCV3-positive samples had a high rate of co-infection with PCV2, PPV6, and PPV7. To study the evolution of the PCV3 in Northeast China, we sequenced the entire genome of 13 strains of PCV3. The results of phylogenetic analyses revealed that PCV3 could be divided into two clades, PCV3a and PCV3b. Interestingly, a G deletion at position 1072 was found in the 1999 nt genome of PCV3-CN2018LN-4 (MH277118). The G deletion terminated replicase protein translation and induced a truncated replicase protein.

**Conclusion:**

These results contribute to the understanding of PCV3 molecular epidemiology and evolution in Northeast China. A new strain of PCV3 with truncated replicase protein was identified.

## Background

Porcine circovirus is a small non-enveloped virus with a circular, single-stranded DNA genome, belonging to the family *Circoviridae* [1]. Porcine circovirus 1 (PCV1) was initially identified in the 1970s as a contaminating agent in pig kidney cells and was considered nonpathogenic for swine [2, 3]. Porcine circovirus type 2 (PCV2), known as an essential pathogen of porcine circovirus-associated disease (PCVAD) clinically manifests as postweaning multisystemic wasting syndrome (PMWS), porcine dermatitis and nephropathy syndrome (PDNS), and reproductive failure, resulting in huge economic losses for the swine industry [4–6]. Currently, a novel circovirus termed porcine circovirus 3 (PCV3), was identified in the United States by metagenomic sequencing. PCV3 is characterized by PDNS, reproductive failure, as well as cardiac and multisystemic inflammation [7, 8]. Subsequently, other researchers found that PCV3 might be associated with congenital tremors (CT) and porcine respiratory disease complex (PRDC) [9, 10]. In addition, several strains of circovirus were found to be associated with different clinical diseases, such as fox circovirus, dog circovirus and duck circovirus [11–13]. Therefore, these data indicate that PCV3 might be associated with pathogenicity in pigs.

Similar to PCV2, PCV3 has a circular single-stranded DNA genome ranging in size from 1999 to 2001 nucleotides, containing two major open reading frames (ORFs), ORF1 and ORF2, which code for a 296 amino acid (aa) replicase protein (rep) and 214 aa capsid protein (cap), respectively [7, 8]. PCV3 is widely circulated across the world, including the United States, China, Italy, Brazil, Korea, German, Denmark, and Spain [14–19]. In addition, increasing evidence has shown that PCV3 co-infections with other pathogens might be associated with increased pathogenicity in pigs [8, 20]. Moreover, the study by Li et al. demonstrated that PCV3 could be divided into two clades using the complete coding sequences [21, 22]. However, a greater number of PCV3 sequences are required to verify the division of PCV3 into different clades. In the present study, we investigated the extent of infection and co-infection of PCV3 in Northeast China. Furthermore, 62 complete coding sequences (13 in this study) were used for phylogenetic analysis and dividing PCV3 into different clades. More importantly, a new strain of PCV3 with a 13 aa deletion in the replicase protein was identified.

## Results

### Characteristics of the PCV3 epidemic in Northeast China

**Fig. 1:**
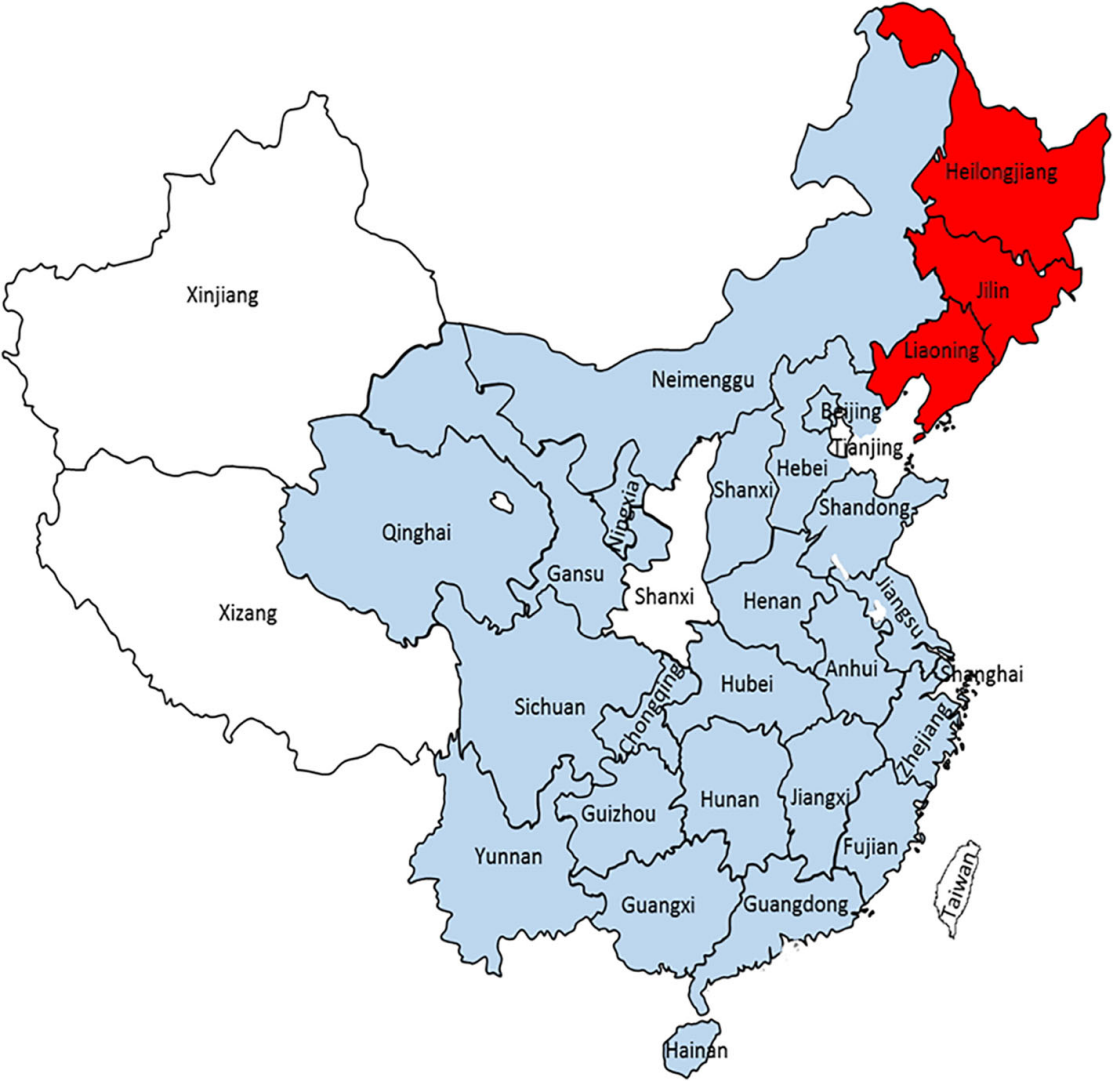
Geographical distributions of PCV3 in China. Red represents the PCV3-positive provinces in this study. Light blue represents the PCV3-positive provinces in previous reports [14, 20, 26, 27]

Of the 105 clinical lung samples obtained from sick pigs in Northeast China, the total PCV3-positive rate was 33.3% (35/105), with 17.8% (8/45) positivity in Heilongjiang, 66.7% (10/15) in Jilin, and 37.8% (17/45) in Liaoning. In addition, both the present and previous studies demonstrate that the presence of PCV3 is extensive in China (Fig. 1).

### PCV3 co-infection with other pathogens

Among the 35 PCV3-positive samples, co-infection of PCV3 with other pathogens, including CSFV, PRRSV, SIV, PRV, PPV2, PPV6, PPV7, TTSuV1, TTSuV2, and PCV2 was analyzed. As shown in Table 1, PCV3 co-infections with PRRSV, PCV2, TTSuV1, TTSuV2, PPV2, PPV6, and PPV7 were detected in 11.4% (4/35), 45.7% (16/35), 34.3% (12/35), 25.7% (9/35), 25.7% (9/35), 60.0% (21/35), and 74.3% (26/35) of the samples, respectively. In contrast, neither CSFV, SIV, nor PRV were detected in the PCV3-positive samples. These data indicate that a high co-infection rate of PCV3 with PCV2, PPV6, and PPV7 exists, which provide valuable information for further study into the pathology of PCV3 in association with PCV2, PPV6, and PPV7.

**Table 1:** Detection of PCV3 co-infections with PRRSV, PCV2, TTSuV1, TTSuV2, PPV2, PPV6, PPV7, CSFV, PRV, and SIV in tissue samples isolated from sick pigs during 2018

Geographical origin	Number of samples	PCV3 positive	Co-infection									
			PRRSV	PCV2	TTSuV1	TTSuV2	PPV2	PPV6	PPV7	CSFV	PRV	SIV
Heilongjiang	45	8/45	3/8	3/8	4/8	2/8	2/8	6/8	6/8	0/8	0/8	0/8
Jilin	15	10/15	0/10	6/10	1/10	2/10	3/10	5/10	5/10	0/10	0/10	0/10
Liaoning	45	17/45	1/17	7/17	7/17	5/17	4/17	10/17	15/17	0/17	0/17	0/17
Northeast in China (total)	105	35/105	4/35	16/35	12/35	9/35	9/35	21/35	26/35	0/35	0/35	0/35

### Sequence comparison and phylogenetic analysis of PCV3

**Fig. 2:**
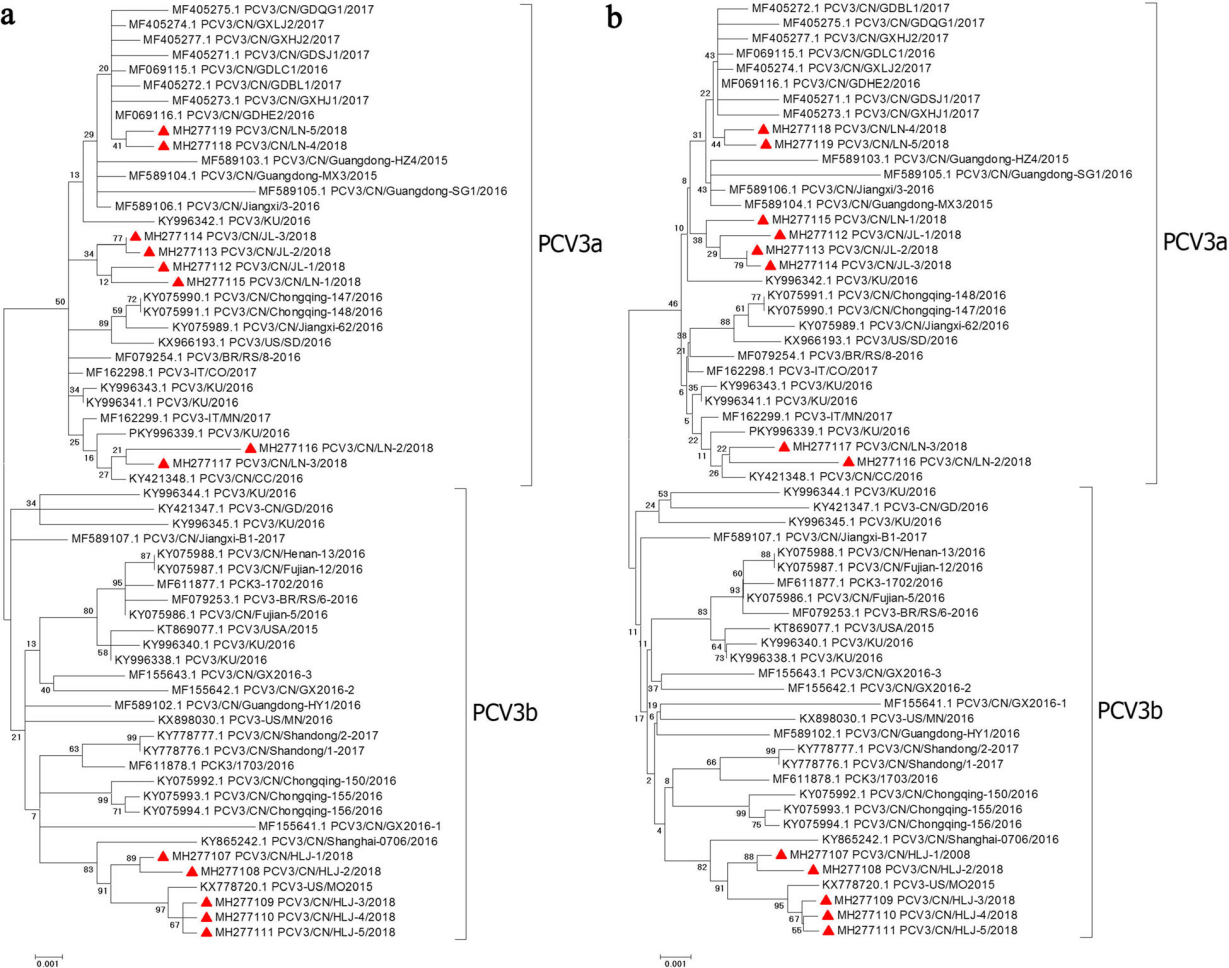
Phylogenetic analysis of the 13 complete PCV3 genomes isolated in this study, together with 49 complete PCV3 genomes obtained from GenBank. The tree was constructed using MEGA version 6.06 with 1000 bootstraps replicates, p-distance model. PCV3 strains are denoted by its accession number at GenBank, isolation year, and country of origin. The 13 strains in this study are indicated with a red triangle. **a** maximum-likelihood method. **b** neighbor joining method

To analyze the genetic relationship of PCV3 strains collected in Northeast China, 13 complete sequences of the PCV3 genome were amplified and sequenced. The complete genome sequences of the 13 samples infected with PCV3 identified in this study were deposited in GenBank under accession numbers MH277107-MH277119. Their genomes were two sizes: 1999 (MH277118) and 2000 nucleotides in length. The alignment of multiple sequences within these 13 PCV3 samples shared 97.9% to 100% and 98.8% to 99.9% nucleotide similarities at the ORF2 and complete genome sequences, respectively. The samples also shared 97.5% to 100% and 97.6% to 99.8% nucleotide similarities with available PCV3 ORF2 and complete genome sequences from the NCBI GenBank, respectively. The method described by Li et al. for dividing clades of PCV3 was used in this study [21, 22]. A total of 49 complete genome sequences were available in the NCBI database, which were used together with the 13 complete genome sequences (MH277107-MH277119) in this study to divide PCV3 into different clades. The NJ and ML tree was performed to reconstruct the phylogenies to evaluate the PCV3 complete genome sequences. This analysis revealed that the PCV3 strains were divided into two clades, PCV3a and PCV3b (Fig. 2). The majority of the PCV3 strains identified in this study were of the PCV3a and PCV3b clades. A phylogenetic analysis based on the complete genomes indicated that the 13 PCV3 strains in this study clustered more closely around the PCV3 strains identified in United States, South Korea, Italy and Southwest China (Fig. 2). The results revealed that the PCV3 variants in Northeast China exhibited a uniform distribution of the different PCV3 strains across the world.

### PCV3 genome with a G deletion induced a truncated replicase protein

**Fig. 3:**
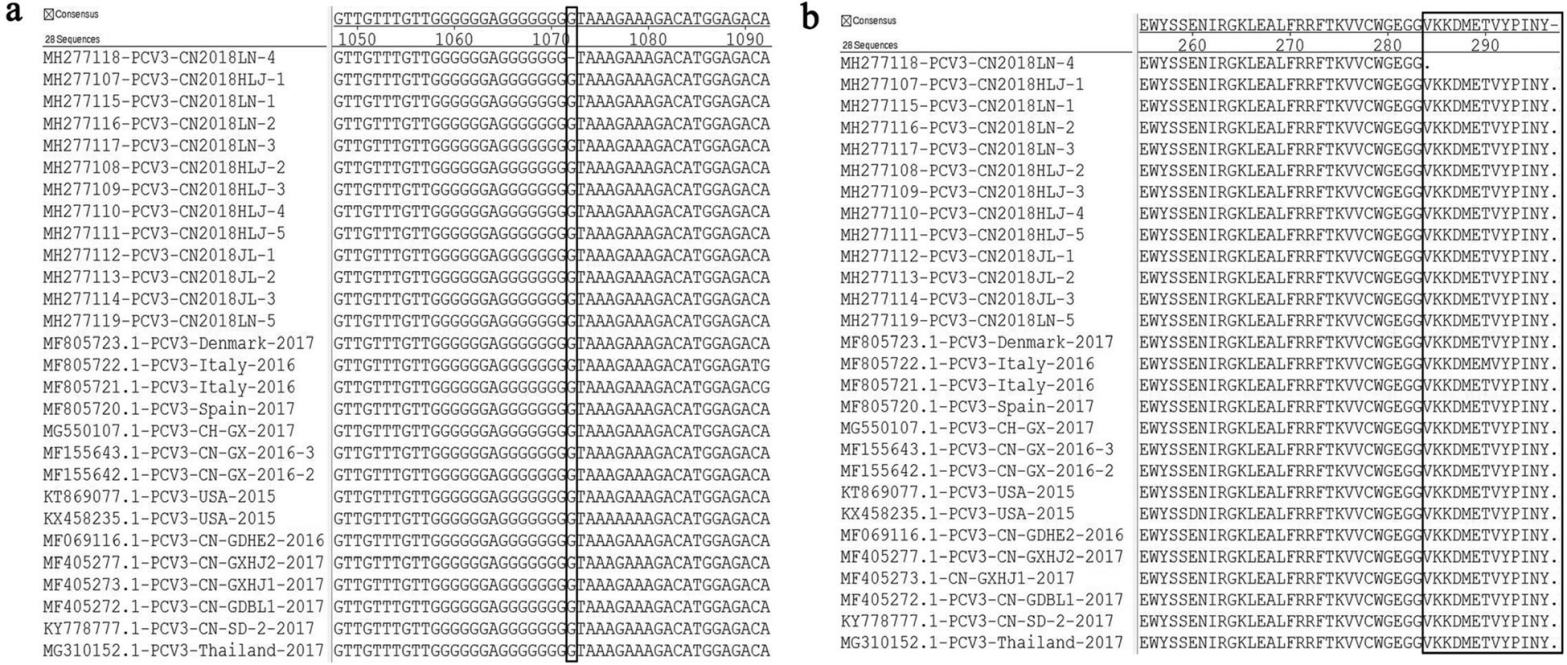
Multiple alignments of the complete PCV3-CN2018LN-4 genome sequences and amino acids of the Rep protein with the other 27 PCV3 strains. (**a**) Complete PCV3 genome sequences between the sites 1048 and 1092. PCV3-CN2018LN-4 contains a G deletion at position 1072 compared with the other PCV3 strains. The deletion of G is indicated by a block. (**b**) PCV3 Rep protein between amino acid sites 255 and 296. There is a 13-amino acid deletion of PCV3-CN2018LN-4 from the amino acid positions 284 to 296. The deletion of 13 amino acids is indicated with a block

Surprisingly, from the 13 complete PCV3 genome sequences in our study, the genome sequences of strain PCV3-CN2018LN-4 (MH277118) had a G deletion at position 1072 compared with the total genome sequences of the PCV3 strains available in the NCBI database (Fig. 3a). The G deletion was found to prematurely terminate Rep protein translation, inducing a truncated Rep protein of 283 aa (Fig. 3b). Thus, the influence of this truncated Rep protein on the pathogenicity of PCV3 requires further study.

## Discussion

Since PCV3 was first identified, PCV3 has been associated with PDNS, CT, reproductive failure, as well as cardiac and multi-systemic inflammation in infected pigs [7, 8]. However, whether PCV3 is capable of inducing a broad range of pathological effects similar to PCV2 requires further study. In this study, the co-infection of PCV3 with other pathogens was investigated using samples isolated from sick pigs. Moreover, PRRSV, PCV2, PRV, CSFV, SIV, TTSuV1, TTSuV2, PPV2, PPV6, and PPV7 were detected in the PCV3-positive tissue samples. Although no co-infections with CSFV, SIV, or PRV were detected in the PCV3-positive tissue samples, a high prevalence of PCV2, PPV6, and PPV7 existed in the PCV3-positive pigs. Previous studies have identified PCV3 and TTSuV1 co-infections in PDNS sows from the United States [8]. Moreover, a high co-infection rate of TTSuV1 and TTSuV2 with PCV3 was detected among clinically healthy sows [23], whereas a co-infection of PCV3 with PRRSV existed in pigs exhibiting severe respiratory disease [20]. Furthermore, a single PCV3 infection was found in pigs with reproductive failure [15]. In the present study, a single PCV3 infection was observed in four tissue of the tissue samples. In addition, this is the first study to report a high prevalence of PPV6 and PPV7 co-infection in PCV3-positive pigs. However, further study is required to elucidate whether PCV3 alone or co-infection with PCV2, PPV6, or PPV7 is involved in the pathogenicity exhibited in pigs. Therefore, there is an urgent need to isolate various PCV3 strains in vitro and evaluate the pathogenicity in pigs with PCV3 infections or co-infections with the above pathogens. In the present study, we attempted to isolate PCV3 in porcine kidney (PK-15) and swine testis (ST) cells; however, PCV3 was not found in either of the cells after four passages. In addition, an infectious molecular clone was constructed using PK-15 cells to isolate PCV3 but it also failed (data not shown). Thus, it is important to explore new approaches that can be used for successful PCV3 isolation.

PCV3 is widely circulated across the world, especially in China, where it has been detected almost provinces (Fig. 1). Recent epidemiological survey showed the positive rate of PCV3 infection between 19.1 and 39.4% in some provinces of China [15, 24–26]. In the present study, the positive rates of PCV3 were 17.8%, 66.7% and 37.8% in Heilongjiang, Jilin, and Liaoning province, respectively. Limited detection of sample numbers may result in the high prevalence in Jilin and low prevalence in Heilongjiang. However, a greater number of samples are required to verify the positive rate of PCV3 in Northeast China. The phylogenetic analysis of the PCV3 genome sequences revealed that the PCV3 strains in this study are closely related to strains isolated in United States (KX778720), South Korea (KY996339), Italy (MF162299), and Southwest China (KY421348). These results indicate that the PCV3 variants in Northeast China have a uniform distribution with different PCV3 strains across the world. A possible reason for this might be the presence of PCV3 infections within swine herds over long periods of time. Previous studies have found that PCV3 infections in China can be traced back to 1996 [27]. In addition, a common recent ancestor analysis suggests that PCV3 lineages have been circulating among swine herds over the past 50 years [26, 28].

The PCV2 strains were divided into different genotypes based on an analysis of the ORF2 gene sequences, from which a distance genotype was calibrated [29]. Fu et al. divided PCV3 was divided into three clades based on the aa codons in ORF2 (24, 27, 77, 104, and 150 aa) [26]. Fux et al. divided PCV3 into a1, a2, b1, and b2 subtypes based on the aa codons in ORF1 (122 aa), ORF2 (24, 27, 77, and 150 aa), and ORF3 (1, 4, and 27 aa) [30]. Subsequently, Li et al. study showed the phylogenetic analysis of PCV3 did not show clear clusters and consistent in different references based on the sequences of the ORF2 gene. But, by ML, maximum clade credibility (MCC), and NJ methods to reconstruct the phylogenies of PCV3 using complete coding sequences, PCV3 could be stably divided into two clades, PCV3a and PCV3b, and further confirmed by principle component analysis (PCA) [21, 22]. Li et al. study provided a comprehensive genotype identification. In the present study, we referred to the methods used by Li et al. to divide the strains of PVC3 into different clades. We used the NJ and ML tree to reconstruct the phylogenies of PCV3 complete genome sequences. Two different trees displayed similar structures in the division of PCV3 into different clades. PCV3 strains could be divided into two clades, PCV3a and PCV3b. Therefore, phylogenetic analysis of complete coding sequences might be considered as PCV3 clade division in the future.

A previously study showed that the PCV3 genome was 1999–2001 nucleotides in length [8, 25, 30]. Moreover, the genome consisted of two inverse ORFs, coding for the Rep (296 aa) and cap (214 aa) proteins, respectively [8]. In this study, the 13 complete PCV3 genomes were 2000 or 1999 nucleotides in length. However, a G deletion at position 1072 of the PCV3-CN2018LN-4 (MH277118) strain was observed, which differs from previous reports. Interestingly, the G deletion at position of 1072 was in the coding region of the Rep protein, as the termination codon, TAA, is situated after the G gene. Thus, the G deletion induced a truncated Rep protein of 283 aa. This finding suggests that two different lengths (296 aa and 283 aa) of the Rep protein exist in PCV3. However, the impact of the truncated Rep protein (283 aa) in PCV3 infections requires further study.

## Conclusions

PCV3 could be divided into two clades using complete coding sequences. A new strain PCV3-CN2018LN-4 (MH277118) had a G deletion in the coding region of the Rep protein and induced a truncated Rep protein of 283 aa, this finding suggests that two different lengths (296 aa and 283 aa) of the Rep protein exist in PCV3. There is a high prevalence of PPV6 and PPV7 co-infection in PCV3-positive pigs.

## Methods

### Tissue samples

In January 2018, a total of 105 lung tissue samples isolated from sick pigs were collected from seven large pig farms in Northeast China, three farms in Heilongjiang, one farm in Jilin, and three farms in Liaoning. All pigs were euthanized by an anesthetic overdose with the pentobarbital (100 mg/kg of body weight) before collecting the samples. Each pig farm consisted of a herd of over 10,000 sows. The age of all sampled pigs ranged from 4 to 16 weeks.

### PCV3 detection

Viral DNA was extracted from the tissue samples using a Tissue DNA Kit (OMEGA Bio-Tek, Georgia, USA) according to the manufacturers’ instructions and tested for PCV3 using PCR, as described previously [9]. Briefly, the detection of PCV3 by PCR was performed in a 25 μL final volume, consisting of 2 μL DNA, 1 μL of each 10 μM primer, 2 μL (2.5 mmol/L) dNTPs, 5 μL PCR buffer, 0.5 μL DNA polymerase (TransGen Biotech, Beijing, China), and ddH_2_O up to 25 μL. The profile of the PCR conditions were as follows: 95 °C for 2 min, 35 cycles of 95 °C for 20 s, 56 °C for 20 s and 72 °C for 20 s, and a final extension at 72 °C for 5 min. The amplified products were analyzed on 1% agarose gels and the positive amplicons were sequenced (Comate Biosciences Co., Jilin, China).

### Co-infection detection

For PCV3-positive tissue samples, viral RNA was extracted using a commercial RNA extraction kit (Sangon Biotech, Shanghai, China) in accordance with the instruction manual. PRRSV, SIV, and CSFV were detected using a one-step reverse transcription PCR Kit (TransGen Biotech, Beijing, China); the primers used for detection were the same as that described previously [31–33]. In addition, the DNA from PCV3-positive tissue samples was selected for the detection of PCV2, TTSuV1, TTSuV2, PPV2, PPV6, PPV7, and PRV, using the same primers as those previously described [34–38].

### PCV3 genome sequencing and phylogenetic analysis

To obtain a full-length sequence of PCV3 for phylogenetic analysis, three pairs of overlapping PCR primers were utilized for whole genome sequencing as previously described [8]. PCR assays were performed using TransGen Biotech DNA polymerase (TransGen Biotech, Beijing, China). The PCR profile conditions were as follows: 95 °C for 2 min, 35 cycles at 95 °C for 20 s, 50 °C for 20 s, and 72 °C for 40 s, with a final extension at 72 °C for 5 min. The PCR products were purified using a Gel Extraction Kit (Bioer Technology, Hangzhou, China) and cloned into a pEASY-Blunt vector (TransGen Biotech, Beijing, China) for sequencing (Comate Biosciences Co., Jilin, China). A total of 13 complete PCV3 genome sequences were obtained in this study and all of the available complete PCV3 genomes from the NCBI GenBank were used for alignment and phylogenetic analysis. Multiple sequence alignments were performed using Lasergene software with the Clustal W program implemented in DNAStar software. A phylogenetic analysis of the complete PCV3 genome was reconstructed using MEGA 6.06 software with the p-distance-based, maximum-likelihood (ML) method and neighbor joining (NJ) method with 1000 bootstrap replicates.

## Abbreviations

A: Alanine
K: Lysine
ORFs: Open reading frames
PCV3: Porcine circovirus type 3
PPV6: Porcine parvovirus 6
PPV7: Porcine parvovirus 7
R: Arginine; aa: amino acid
rep: Replicase protein
V: Valine
